# Effect of Adding Curcumin on the Properties of Linseed Oil Organogels Used as Fat Replacers in Pâtés

**DOI:** 10.3390/antiox9080735

**Published:** 2020-08-11

**Authors:** Patricia Ramírez-Carrasco, Javier Paredes-Toledo, Patricio Romero-Hasler, Eduardo Soto-Bustamante, Paulo Díaz-Calderón, Paz Robert, Begoña Giménez

**Affiliations:** 1 Departamento de Ciencia de los Alimentos y Tecnología Química, Facultad de Ciencias Químicas y Farmacéuticas, Universidad de Chile, Santos Dumont 964, Independencia, 8380494 Santiago, Chile; patricia.ramirez@ug.uchile.cl (P.R.-C.); proberts@uchile.cl (P.R.); 2 Departamento de Ciencia y Tecnología de los Alimentos, Facultad Tecnológica, Universidad de Santiago de Chile, Av. Ecuador 3769, Estación Central, 9170124 Santiago, Chile; javier.paredes@usach.cl; 3 Departamento de Química Orgánica y Fisicoquímica, Facultad de Ciencias Químicas y Farmacéuticas, Universidad de Chile, Santos Dumont 964, Independencia, 8380494 Santiago, Chile; patricio.romero@hotmail.com (P.R.-H.); esoto@ciq.uchile.cl (E.S.-B.); 4 Biopolymer Research & Engineering Laboratory (BIOPREL), Escuela de Nutrición y Dietética, Facultad de Medicina, Universidad de los Andes, Avda. Monseñor Álvaro del Portillo 12,455, Las Condes, 7620001 Santiago, Chile; pdiaz@uandes.cl; 5 Centro de Investigación e Innovación Biomédica, Facultad de Medicina, Universidad de los Andes, Avda. Monseñor Álvaro del Portillo 12,455, Las Condes, 7620001 Santiago, Chile

**Keywords:** beeswax, linseed oil, curcumin, pâté, oxidative stability

## Abstract

Beeswax-based organogels were formulated with linseed oil and curcumin according to a statistical design to increase the oxidative stability of spreadable meat products (pâté) where these organogels (OGCur) were incorporated as fat substitutes. The organogels obtained under optimal conditions (9.12% beeswax, 0.54% curcumin) showed a mechanical strength similar to pork backfat determined by back extrusion and high oil binding capacity (OBC; over 90%). The incorporation of curcumin at this concentration did not lead to any change in the arrangement of the crystal network, OBC, and mechanical, thermal, or rheological properties of the organogels. Beeswax organogels with and without curcumin, with a β’ orthorhombic subcell structure, showed a predominant elastic behavior and a melting event wider and shifted to lower temperatures than pure beeswax, suggesting a plasticizer effect of the oil in the wax crystals. The oxidative stability of the organogels under accelerated oxidation conditions increased due to the incorporation of curcumin. A decrease in the curcumin content was found from day 4 at 60 °C, together with a significantly lower formation of both peroxides and malonaldehyde. When pork backfat was partially or totally replaced by OGCur in pâtés, a noticeable protective effect of curcumin against lipid oxidation was found during chilled storage

## 1. Introduction

Organogelation is one of the most novel and promising oil structuring technologies that has recently received increased attention in several fields including cosmetic, pharmaceutical and food science [1,2,3]. Organogels can be defined as semisolid systems where an organic liquid (oil) is entrapped in a three-dimensional network of lipophilic solids (organogelators) [4]. Since organogelation enables the formation of self-standing, three-dimensional gel networks with viscoelastic properties from liquid oils, without changing the fatty acid composition [4], this approach has been employed for the replacement of solid animal fat by vegetable and/or marine liquid oils, rich in polyunsaturated fatty acids, in the development of foods with a healthier lipid profile, such as spreads, bakeries, confectioneries, and dairy and meat products [5].

In this context, organogels based on vegetable and/or marine oils have been recently applied with promising results for the replacement of animal fat in meat products, such as frankfurters or sausages, meat patties or pâté, since these products make a large contribution to the dietary intake of fat and saturated fatty acids [5,6]. Linseed oil has been widely used in the development of healthier and/or functional foods since it is one of the greatest sources of α-linolenic acid, essential fatty acid to which several human health promoting effects have been attributed, and the precursor of the omega-3 long-chain polyunsaturated fatty acids in the organism [7]. This vegetable oil has also been incorporated into organogels, alone or in combination with other oils [8,9,10,11,12]. Different organogelators have been used for this purpose, mostly ethylcellulose, but also plant sterols, mixtures of monoglycerides and phytosterols, and natural waxes [5]. The use of beeswax as structuring agent for fat replacement in meat products is still scarce [9,10,11,12,13], and varying results have been obtained as a function of the reformulated meat product. To the best of our knowledge, there is only one study that focused on the replacement of pork backfat by beeswax-based organogels in pâté, where the technological behavior, color and texture of the reformulated products were similar to the sample formulated with pork backfat [10]. Although the replacement of animal fat by organogels based on oils rich in polyunsaturated fatty acids, such as linseed oil, may promote the prevention of several diet-related chronic diseases, such as cardiovascular diseases, due to the reduction in saturated fatty acids and the increase in polyunsaturated fatty acids [14]; this may also lead to a decrease in oxidative stability of the meat products [10,13] since polyunsaturated fatty acids are very prone to oxidation. Furthermore, the oil structuring by direct dispersion of the organogelators in the oil phase involves continuous agitation at high temperature that may promote lipid oxidation. Autoxidation is the main mechanism involved in lipid oxidation in meat and meat products, which depends on several intrinsic and extrinsic factors. Fatty acid composition is one of the most important intrinsic factors, since they are the main substrate for lipid oxidation, and the oxidative stability has been related to the number and position of unsaturations, fatty acid conformation, as well as the chain length [15]. Incorporation of lipophilic antioxidants into the organogel matrix may be the most effective strategy to prevent lipid oxidation during both the preparation process and storage of the organogel, as well as to increase the oxidative stability of the meat products where these organogels are used as fat substitutes. However, the incorporation of antioxidants in organogel formulations is still scarce [12,16,17], but this is a relevant topic that is directly associated with the stability of these systems and their capacity to increase both the nutritional value and shelf-life of food products where they are included [2].

Curcumin is a biologically active polyphenol from the turmeric rhizomes consisting of two aromatic rings bearing two hydroxyl and two methoxyl groups, which has several biological properties such as anti-inflammatory, anticancer, antibacterial and antioxidant [18]. Curcumin has been incorporated in different liquid edible oils to successfully prevent lipid oxidation, such as fish oil [19], sunflower oil [20,21] and soybean oil [22]. In two recent studies, curcumin has also been incorporated to organogels based on corn oil and a mixture of olive, linseed and fish oil [12,23] to increase their oxidative stability. However, the possible influence of curcumin, not only on the oxidative stability, but also on the arrangement of the crystalline network as well as thermal and rheological properties should be elucidated.

The objective of this study was to formulate beeswax linseed organogels with curcumin according to a statistical design, to increase the oxidative stability of spreadable meat products (pâté), where organogels were incorporated as fat substitutes. Furthermore, the influence of curcumin on the arrangement of the crystalline network, oil binding capacity, and mechanical, thermal and rheological properties of the organogels was evaluated.

## 2. Materials and Methods

### 2.1. Materials

The linseed oil (fatty acid composition: 57.02 ± 0.25% C18:3 n-3, 14.51 ± 0.11% C18:2 n-6, 19.64 ± 0.12% C18:1, 5.23 ± 0.01% C16:0 and 3.61 ± 0.02% C18:0) was purchased from Nutra Andes Ltda. (Valparaíso, Chile). Beeswax (Ceratech 8104) was obtained from Coprin S.A. (Santiago, Chile), and curcumin was purchased from Xi’an Xin Sheng Bio-Chem Co. (Xi’an, China). Pork meat, liver and backfat were purchased in a local market.

### 2.2. Methods

#### 2.2.1. Preparation of Linseed Oleogels

A central composite + start design (12 runs with 3 central points) was applied to prepare linseed organogels. The concentration of beeswax (5–12% *w*/*w*) and curcumin (0.1–0.5% *w*/*w*) were the independent variables, whereas the mechanical strength, the oil binding capacity (OBC) after 24 h at 25 °C and the oxidative stability evaluated by the induction period (IP) under accelerated oxidation conditions (Rancimat) were the response variables. The experiments were performed randomly to avoid systematic bias. Data were fit to a second-order regression model (Equation (1)):

(1) $$Y=b_{0}+\;\overset{2}{\underset{i=1}{\sum }}b_{i}X_{i}+\overset{2}{\underset{i=1}{\sum }}b_{ii}X_{i}^{2}+\overset{1}{\underset{i=1}{\sum }}\overset{2}{\underset{j=i+1}{\sum }}b_{ij}X_{i}X_{j}$$

where *Y* was the estimated response (mechanical strength, OBC or IP), subscript *i* and *j* ranged from 1 to 2 (*n* = 2), b_0_ was the intercept term, *b_i_*, *b_ii_*, and *b_ij_* values were the linear, quadratic and interaction coefficients, respectively, and *X_i_* and *X_j_* were the levels of the independent variables.

Non-significant terms were removed from the equation, except in the case of linear forms of concentration of beeswax or concentration of curcumin, since they are essential elements of the mathematical model. Statgraphics Centurion XVI software (Statistical Graphics Corporation, Warrenton, VA, USA) was used to perform the analysis of variance (ANOVA) and lack-of-fit test, as well as to determine the regression coefficients. Response surface methodology (RSM) was applied to determine the optimal conditions for each independent variable. Optimization was performed using the desirability function. While OBC and IP were maximized, the mechanical strength was fixed to the mechanical strength obtained for pork backfat by back extrusion.

The organogel (100 g) was formulated as follows: beeswax (5–12 g) was dispersed in linseed oil (74.9–67.5 g) at 70 °C under stirring (700 rpm). Curcumin (0.1–0.5 g) was incorporated in the remaining linseed oil (20 g) and heated until 140 °C, according to Yu et al. [24] to ensure total dissolution. Once this temperature was achieved, the curcumin in oil was added to the mixture of beeswax and linseed oil and stirred for 1 min. The resultant solution was maintained at room temperature for 30 min, and subsequently at 4 °C until further analysis.

#### 2.2.2. Characterization of Organogels Obtained under Optimal Conditions

The organogels with curcumin obtained under optimal conditions (OGCur) were characterized according to the following methodologies. Furthermore, organogels without curcumin and with the optimal beeswax concentration (control, OGCtrl) were formulated as described in Section 2.2.1 to evaluate the effect of the incorporation of curcumin on the organogel properties.

##### Polarized Light Microscopy

A polarized light microscope (DLMP, Leica, Wetzlar, Germany), equipped with a heating stage (HCS302/STC200, Instec, Boulder, CO, USA), was used to study the temperature-dependent formation of the organogels. The organogels (OGCur and OGCtrl) were placed onto microscope slides and covered with a cover glass. The microscope was coupled with a Canon 750D camera, used for obtaining the photomicrographs. The heating (from 20 °C to 80 °C) and cooling ramps (from 80 °C to 20 °C) were performed at 2 °C/min. The micrographs of preliminary studies, where beeswax and curcumin were dissolved by heating at 70 °C (over the melting point of the beeswax) instead of 140 °C were also shown.

##### Mechanical Strength

The mechanical strength was determined by back extrusion [25], with a texturometer (BDO-FDO.5T5, Zwick/Roell, Ulm, Germany), equipped with a 5 kg load cell and a 10 mm diameter cylindrical probe. The organogels (OGCur and OGCtrl) were poured down in 16 mm diameter tubes, maintained at room temperature for 30 min and stored overnight at 4 °C. The samples were penetrated until 30 mm at 1.5 mms^−1^, and the mechanical strength (N) was expressed as the average force of the last fourth part of the test.

The mechanical strength of pork backfat was also determined by back extrusion. Pork backfat from ten different animals was chopped (3250 rpm, 5 min) and melted (500 rpm, 100 °C, 20 min) in a food processor (Thermomix TM31, Vorwek, Wuppertal, Germany). Then, the pork backfat was filtered through a gauze and placed in 16 mm diameter tubes, maintained for 30 min at room temperature and stored overnight at 4 °C. The back extrusion test was performed as described for organogels.

##### Oil Binding Capacity

OBC of OGCur and OGCtrl was measured according to da Silva et al. [26]. Cylindrical samples (1 cm length × 1 cm diameter) were weighted and placed on filter papers (Whatman N°1) supported by a Petri dish. The oil loss was determined by weighting the Petri dish and the filter paper before placing the samples on them, and after 1 day and 7 days of storage at 25 °C. The OBC was calculated according to the following equation (Equation (2)):

(2) $$\text{OBC }\left(\%\right)=100-\frac{\left({\text{W}}_{\text{tf}}-{\text{W}}_{\text{t}0}\right)}{{\text{W}}_{\text{s}}}\times 100$$

where, W_tf_ is the weight (g) of Petri dish and filter paper after 1 or 7 days, W_t0_ is the weight (g) of Petri dish and filter paper before the samples were placed on, and W_S_ (g) is the weight of the organogel.

##### Induction Period

The IP of OGCur and OGCtrl was determined under accelerated oxidation conditions by using a Rancimat (892 equipment, Metrohm Ltd., Switzerland) at 100 °C with an air flow of 20 L/h. The protection factor (PF) was calculated according to the following equation (Equation (3)):

(3) $$\text{PF}=\frac{\text{IP OGCur }\left(\text{h}\right)}{\text{IP OGCtrl }\left(\text{h}\right)}$$

##### Thermal Analysis

The thermal analysis of beeswax and organogels (OGCur and OGCtrl) was performed with a differential scanning calorimeter (TA Q20, TA Instruments, New Castle, DE, USA). Samples (5–10 mg) were placed in sealed aluminum pans (TA T-Zero pans) under nitrogen purge (50 mL/min), and melted at 80 °C in the DSC chamber to erase the thermal history. Afterwards, modulated differential scanning calorimetry (MDSC) was measured from −40 °C to 120 °C at a rate of 1.5 °C/s, with a modulation regime of 1.5 °C every 90 s. The thermal properties of the samples were analyzed by the resulting heat flow thermograms using the TA Instruments Universal Analysis 2000 software (New Castel, DE, USA). The DSC was calibrated using Indium (156.6 °C) as standard and a ramp rate of 5 °C/min. As control, three different temperature standards were used: benzophenone (ME18870; melting point 47.9 ± 0.2 °C), benzoic acid (ME18555; melting point 122.3 ± 0.2 °C) and caffeine (ME18872; melting point 236.0 ± 0.3 °C).

##### X-ray Diffraction Analysis

The X-ray diffraction (XRD) patterns of beeswax and organogels (OGCur and OGCtrl) were collected at small (SAXD) and wide angles (WAXD) at 30 °C, with a Stoe Stadi P diffractometer (Stoe, Darmstadt, Germany), using a long fine focus CuKα source at 40 kV and 30 mA monochromated with a Ge 111 curved crystal (λ = 1.54060). For temperature dependent measurements, only the small-angle region between 1° and 6° in 2θ and the wide-angle region between 20° and 26° in 2θ were measured, under a cooling ramp from 70 °C to 30 °C at a rate of 0.1 °C/min. Each measurement at small and wide angles took 5 min, equivalent to 0.5 °C temperature span. The samples were confined in 1.0 mm diameter Lindemann glass capillaries and mounted on a modified hot stage (HCS-302, Instec, Boulder, CO, USA) for temperature dependent analysis. The detector used was a STOE linear PSD with a 4.3° angular range. The calibration was performed using silver behenate for low angles and NIST 640c silicon for the wide-angle region.

##### Rheological Analysis

Steady and dynamic rheological measurements were performed on OGCur and OGCtrl by using a rheometer (HR-2 Discovery Hybrid Rheometer, TA Instruments, New Castle, DE, USA), with a sandblasted parallel-plate geometry (40 mm diameter, 1 mm gap). Strain amplitude sweeps were performed to determine the linear viscoelastic region (LVR) by varying the shear strain (γ) from 0.01% to 20%, at 6.28 rad/s and 25 °C. Frequency sweeps were performed from 0.1 to 100 rad/s, at γ = 0.01% (within the LVR) and 25 °C. Finally, temperature sweeps were performed to evaluate the thermal stability of the organogels from 5 °C to 70 °C, at a linear heating rate of 2 °C/min, a shear strain of 0.01% and an angular frequency of 6.28 rad/s.

##### Long-Term Oxidation Studies

The oxidative stability of the organogels (OGCur and OGCtrl) was evaluated under accelerated conditions (60 ± 1 °C). Melted organogels (4 g) were placed in tubes (10 mL) with screw cap. Samples were removed in triplicate at specific time intervals to determine peroxide value, thiobarbituric acid reactive substances (TBARs), and the remaining curcumin content.

Peroxide value was determined according to AOCS Cd 8-53 [27], and the results were expressed as meq O_2_/kg organogel. TBARs were determined according to Gómez-Estaca et al. [10]. Organogel (1 g, finely chopped) was mixed with 1 mL of BHT (1% *w*/*v*) and 8 mL of TBARs reagent (15% *w/v* trichloroacetic acid and 0.0375% *w/v* 2-thiobarbituric acid in 0.25 N HCl). The mixture was heated at 95 °C for 15 min and cooled down to room temperature in an ice bath. Hexane (4 mL) and ammonium sulphate 4 M (4 mL) were added to the mixture, vortexed and centrifuged at 8000× *g* and 10 °C for 30 min. The absorbance of the supernatant was measured at 532 nm and the results were expressed as mg of malondialdehyde (MDA)/kg organogel, according to a calibration curve obtained with 1,1,3,3 tetraethoxypropane (0–31 µM; *R*^2^ = 0.99).

The remaining content of curcumin was determined by HPLC according to Jayaprakasha et al. [28]. Organogel (0.5 g) was mixed with petroleum ether (5 mL) and vortexed until it was dissolved. Then, 10 mL of methanol:water (90:10 *v*/*v*) were added and the mixture was centrifuged (8000× *g*, 10 °C, 20 min). The methanolic phase was injected in a Merck Hitachi L-6200 pump equipped with a photodiode-array detector (Waters 996, Milford, MA, USA) and a C18 column (5 μm, 4.6 mm i.d. × 250 mm, Perkin Elmer, UK). Curcumin was quantified using a curcumin standard calibration curve (5–125 μg/mL; *R*^2^ = 0.99).

#### 2.2.3. Oxidative Stability and Fatty Acid Profile of Spreadable Meat Products (pâté)

##### Formulation of Spreadable Meat Products (pâté)

Five pâtés with the same content of fat (15 g/100 g) were formulated according to Gómez-Estaca et al. [10] (Supplementary Table S1). Pork backfat was used as fat ingredient in the control samples (C). Total or partial replacement of pork backfat with OGCur or OGCtrl was performed. The meat batters were prepared using a food processor (Thermomix TM31, Vorwek, Wuppertal, Germany). The ingredients used, pork milled meat, pork liver, pork backfat or organogel, and a mixture of additives previously dissolved in water (sodium chloride, milk powder, sodium caseinate, tripolyphosphate, and species) were added sequentially to the food processor, and the final mixture was homogenized at 4400 rpm until a uniform batter was obtained. The final temperature of meat batters was below 12 °C in all cases. The meat batter was poured down in plastic containers (60 cm^3^) and heated in a water bath until the thermal center achieved 80 °C for 5 min (temperature monitored with a thermocouple). Afterwards, the samples were cooled down in an ice bath and stored at 4 °C until analysis.

##### Fatty Acid Profile of Spreadable Meat Products (pâté)

After lipid extraction from pâté [29], fatty acid methyl esters (FAMEs) were prepared according to UNE-EN ISO 5509:2001 [30] and analyzed in a gas chromatograph (7890B, Agilent, Santiago, Chile), with a fused-silica capillary column (HP-88, 100 m × 0.25 mm i.d. × 0.20 µm film thickness) and a flame ionization detector. The initial oven temperature (180 °C) was held for 20 min, increased to 215 °C (2 °C/min), and held for 15 min. The hydrogen flow was 1 mL/min, and the injector and detector temperatures were 250 °C. The injection volume was 0.5 µL. FAME standards (Sigma-Aldrich, Santiago, Chile) were used to identify the fatty acids, and margaric acid was used as internal standard (Nu-Chek Prep. Inc., Elysian, MN, USA).

##### Thiobarbituric Acid Reactive Substances (TBARs) of Spreadable Meat Products (pâté)

Samples (in triplicate) were removed at specific time intervals over 15 days at 4 °C to determine TBARs according to Delgado-Pando et al. [31]. Briefly, 4 g of each sample were added to a mixture of 1 mL of distilled water and 10 mL of trichloroacetic acid (10% *w*/*v*), and vortexed until complete homogenization. Afterwards, 2-thiobarbituric acid (5 mL, 20 mM) was added to the mixture and the tubes were centrifuged at 8000× *g* and 4 °C for 10 min. The supernatants were stored in darkness at 20 ± 2 °C for 20 h, and the absorbance was measured at 532 nm. The results were expressed as mg malonaldehyde/kg of sample, according to a calibration curve obtained with 1,1,3,3 tetraethoxypropane (0–31 µM; *R*^2^ = 0.99).

#### 2.2.4. Statistical Analysis

All the determinations were performed in triplicate. Analysis of variance (ANOVA) and Tukey’s multiple range tests were performed to determine statistical differences among samples, using Statgraphics Centurion XVI software (Statistical Graphics Corporation, Warrenton, VA, USA). The level of significance was set for *p* < 0.05.

## 3. Results

### 3.1. Formulation of Linseed Organogels with Curcumin

The experimental design and the ANOVA for the formulation of linseed organogels with curcumin are shown in supplementary material (Table S2). Only the linear form of beeswax concentration was significant for the mechanical strength (*p* < 0.05), which varied between 1.14 N and 34.9 N. The mathematical model, after the non-significant terms were removed, explained 97.1% of the variability (*R*^2^ adjusted for degrees of freedom) in mechanical strength, with residual values below 5.0. Although a lack of fit may occur when quadratic and cross-product forms are removed from the model [32], the model was adequate to describe the observed data since the lack of fit was not significant (*p* > 0.05). In the case of OBC (which varied between 42.1% and 95.8%), only the linear and quadratic forms of beeswax concentration were significant (*p* < 0.05); whereas only the linear and quadratic forms of curcumin concentration were significant (*p* < 0.05) on IP of the organogels (which varied from 6.17 h to 9.39 h). The model explained 98.4% and 90.2% of the variability (*R*^2^ adjusted for degrees of freedom) in OBC and IP, respectively. The lack of fit was not significant (*p* > 0.05) in any case, and the residual values were below 5.0.

**Figure 1:**
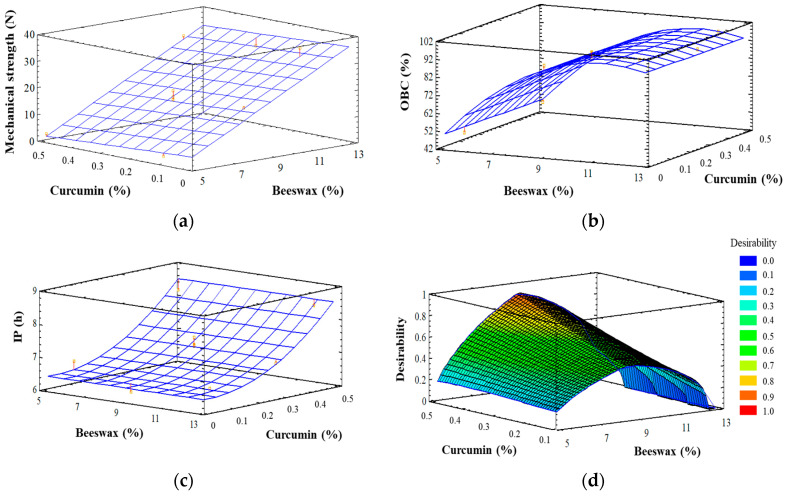
RSM graphics showing the combined effect of concentration of beeswax and curcumin in OGCur on (**a**) mechanical strength, (**b**) oil binding capacity (OBC), (**c**) induction period (IP), (**d**) desirability function.

Figure 1 shows the response surface graphics obtained for mechanical strength (1a), OBC (1b) and IP (1c). According to this figure, the higher the beeswax concentration the higher the mechanical strength, in the range of concentrations studied (Figure 1a). In contrast, the OBC of the organogels increased with increasing concentrations of beeswax until around 11%, and further increases in beeswax concentration did not improve the OBC of the organogels (Figure 1b). The solid concentration and the size of the crystals formed increase with increasing concentrations of wax, leading to a decrease in the proportion of empty space as well as to a more efficient oil entrapment in the matrix, resulting in both higher mechanical strength and [33,34]. Furthermore, the increase in curcumin concentration did not have any significant effect either on the mechanical strength (Figure 1a) or on the OBC (Figure 1b) of the organogels, suggesting that curcumin did not interfere in the arrangement of the crystal network. Furthermore, the higher the curcumin concentration the higher the IP of the organogel, in the range of concentrations studied, whereas the beeswax concentration did not show any effect on IP (Figure 1c). Curcumin is an effective chain breaking antioxidant [35], inhibiting the formation of secondary oxidation products that are determined by Rancimat.

The desirability function was applied to maximize both OBC and IP, whereas the goal for mechanical strength was the value obtained for pork backfat by back extrusion (18.39 ± 0.51 N), since the organogel will be used as a replacement of pork backfat. The highest value for desirability was obtained with a beeswax concentration of 9.12% (*w*/*w*) and a curcumin concentration of 0.54% (*w*/*w*) (Figure 1d). The values predicted by the model for the response variables under these optimal conditions were 91.1%, 8.94 h, and 18.4 N for OBC, IP, and mechanical strength, respectively.

### 3.2. Characterization of Organogels Obtained under Optimal Conditions

#### 3.2.1. Polarized Light Microscopy

Polarized light microscopy (PLM) is a valuable tool to investigate anisotropic materials thanks to their birefringent behavior. Oleogels are known to be composed of a crystalline matrix within a dispersing oil, and this crystalline matrix (beeswax crystals in this case) can be observed under polarized light.

**Figure 2:**
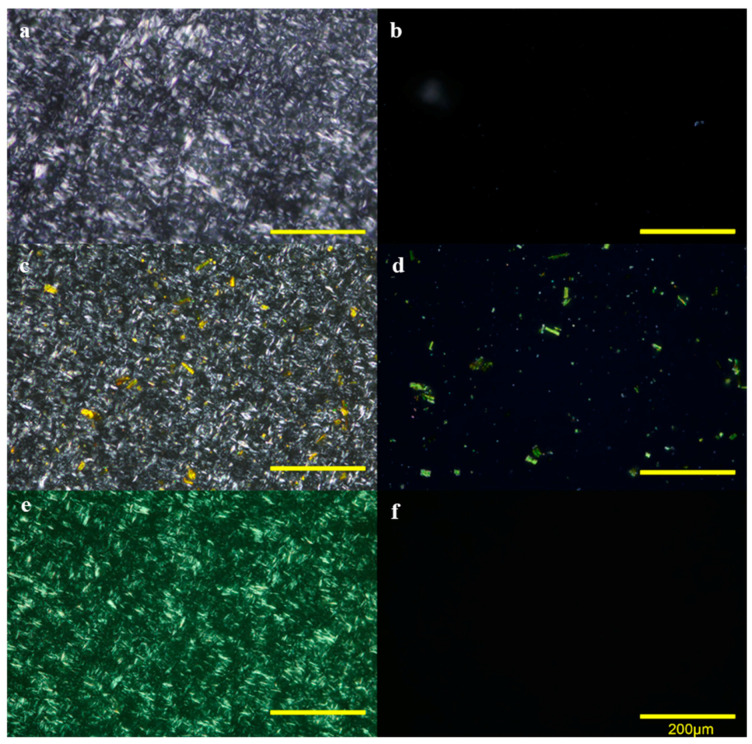
(**a**,**b**) PLM micrographs for OGCtrl at 20 °C (**a**) and at 80 °C (**b**). (**c**,**d**) PLM micrographs for OGCur, with curcumin dissolved at 70 °C ((**c**): organogel at 20 °C; (**d**): organogel at 80 °C). (**e**) and (**f**) PLM micrographs for OGCur, with curcumin dissolved at 140 °C (**e**): organogel at 20 °C; (**f**): organogel at 80 °C).

This study considered the addition of curcumin (0.54% *w*/*w*) as antioxidant in the organogel matrix. Both the gel formation as well as the proper dissolution of curcumin were evaluated by PLM. As it is already known, the melting temperature of curcumin is 175.1 °C [36], while the melting temperature of the beeswax is as low as 61.7 °C (Table 1). Preliminary studies, where beeswax and curcumin were dissolved by heating over the melting point of the beeswax (70 °C), were not successful. Given the high melting point of curcumin, this was intended to dissolve in the linseed oil by heating at 140 °C to ensure its complete dissolution and after that, it was mixed with the beeswax. According to Yu et al. [24], curcumin solubility in oils increased by heating at 140 °C, reporting solubility values over 0.5% (*w*/*v*) in vegetable oils such as corn and canola oil. The micrographs of beeswax organogels with curcumin, prepared both at 70 °C and 140 °C, are shown in Figure 2. Remaining crystals of curcumin were found in organogels when curcumin was dissolved at 70 °C, both at 20 °C (together with beeswax crystals; Figure 2c), and at 80 °C (once the beeswax crystals were melted; Figure 2d). However, when curcumin was dissolved at 140 °C, curcumin crystals at the isotropic state were not found in the organogels (Figure 2e,f).

PLM also provides information about the crystal morphology of the organogels. Fresh prepared samples with and without curcumin (OGCur and OGCtrl, respectively) did not show significant differences under PLM (Figure 2a,e), indicating that the antioxidant was well distributed within the organogel matrix. Both organogels showed needle-like crystals of about 15 µm in average. Dassanayake et al. [37] reported that this fiber-like structure has the ability to form strong gelling networks. The long needle crystals enable to entrap large volumes of liquid oils in the crystalline network. Different crystal morphologies have been reported for beeswax depending on the wax concentration and the type of oil used in the organogel formulation [38,39]. Thus, needle-like structures were also reported in 10% beeswax organogels of olive oil [40], whereas spherulites and spherical agglomerates were found in 10% beeswax organogels of canola, soybean, sunflower, corn, or safflower oil [39].

#### 3.2.2. Mechanical Strength, OBC and IP

Table 1 shows the mechanical strength, OBC and IP of OGCur and OGCtrl. Although curcumin was added in OGCur at 0.54 g/100g, the curcumin content in the organogel matrix was 0.34 ± 0.01 g/100 g, possibly due to the heating (140 °C) during the preparation process of the organogels [41]. The mechanical strength of OGCur was 19.51 ± 0.77 N, close to the goal value obtained for pork backfat (18.39 ± 0.51 N; *p* < 0.05). Moghtadei et al. [13] obtained similar values of back extrusion force (close to 22 N) for sesame oil organogels based on beeswax (10% *w*/*w*) and cooled at 4 °C. Moreover, these authors compared the mechanical force of extracted fats from beef flank and shank with that of sesame oil organogels obtained with different beeswax concentrations and cooled at 4 and 25 °C, and these organogels with 10% of beeswax cooled at 4 °C had the most similar back extrusion force to animal fat (18 N and 21 N for shank and flank fat, respectively).

OBC is an important characteristic in organogels since it represents the strength and nature of the organogelator-oil interactions, especially when the organogels are subjected to mechanical stress [33]. As it is shown in Table 1, OGCur showed OBC values over 90% at day 1, similar to the values predicted by the model (91.1%). Öğütcü and Yilmaz [40], reported higher OBC values (>99%) in walnut organogels based on beeswax (3–10%). However, OBC was determined by drainage of the excess oil after the samples were centrifuged in that study. OBC of OGCur and OGCtrl decreased about 10% after 7 days of storage at 25 °C, showing values around 80%. Therefore, the greatest oil loss occurred during the first day of storage at 25 °C, and, although the oil continued to migrate out of the gel, the oil loss slowed down with storage time. As shown in Table 1, neither the mechanical strength nor the OBC values at days 1 and 7 of storage at 25 °C were significantly affected by the incorporation of curcumin in the organogel, suggesting that curcumin did not interfere with the formation of the tridimensional network that is mainly stabilized by van der Waals forces [42]. Similarly, the incorporation of β-carotene (0.01% *w*/*w*) in beeswax organogels (8% *w*/*w*) did not influence their OBC, but the addition of the bioactive compound led to a higher oil retention in organogels with lower contents of beeswax (2% and 4% *w*/*w*) [16].

Regarding IP measured by Rancimat, the incorporation of curcumin into the organogel matrix significantly (*p* < 0.05) increased IP values, leading to a protection factor of 2.24. Therefore, curcumin effectively protected linseed oil from lipid oxidation, delaying the propagation stage of lipid oxidation and leading to a decrease of the formation of secondary oxidation compounds measured by induction period.

#### 3.2.3. Thermal Analysis

**Figure 3:**
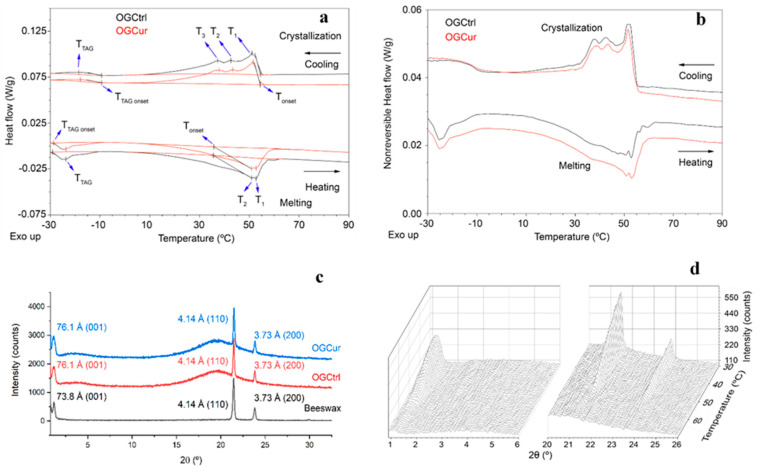
(**a**) MDSC thermograms (total heat flow) for OGCur and OGCtrl. (**b**) MDSC thermograms (non-reversible heat flow) for OGCur and OGCtrl. (**c**) XRD patterns at 30 °C for beeswax (black line), OGCur (blue line) and OGCtrl (red line). (**d**) XRD pattern as a function of temperature during cooling for OGCur.

Table 1 and Figure 3a,b present the data obtained from MDSC by heating and cooling at 1.5 °C/min for the beeswax and the organogels (OGCur and OGCtrl). The obtained values from the X-ray measurements (T_gel_Xray_) were also included in Table 1 for comparative purpose. The melting profile of beeswax showed just one broad melting peak which can be split into three signals at 61.7 °C, 60.2 °C and 51.1 °C respectively (T_1_, T_2_ and T_3_, Table 1; Supplementary Figure S1a). This thermogram is in relatively good agreement with those reported for beeswax in the literature [43]. The presence of this broad melting peak is attributed to the heterogeneous chemical composition of the beeswax [33]. Doan et al. [44] collected the chemical composition reported by different authors for different beeswaxes. Although wax esters were the predominant component in all of them, followed by fatty acids and n-alkanes, along with a small fraction of fatty alcohols, differences in the percentage of these primary groups of components were observed among the different beeswaxes. These differences among samples may lead to differences in the thermal behavior. Furthermore, at least five different melting events were found in the nonreversible heat flow curves of the MDSC experiments for beeswax (Supplementary Figure S1b, black curve), supporting its complex composition.

Two exothermal transitions were found in both OGCur and OGCtrl (Figure 3a,b). One of them at around 35–52 °C, corresponding to the melting of the pure beeswax but wider and shifted to lower temperatures (10 °C) than that found in pure beeswax (Supplementary Figure S1a), suggesting a plasticizer effect of the oil in the wax crystals, which significantly affected the beeswax crystallization [39]. At lower temperatures, another melting event was found (T_TAG_, Table 1), which agrees with the melting/crystallization of the triglycerides of linseed oil, as described by Toro-Vázquez et al. [45]. The total enthalpy for the beeswax was 161.9 J g^−1^ (melting) and 153.5 J g^−1^ (crystallization) (Table 1), in agreement with values reported in the literature [46]. The organogels showed about a 10% of these enthalpy values, in accordance with the beeswax concentration used in the preparation of both organogels (9.12%; Section 3.1.). Remarkable differences were not found between OGCur and OGCtrl in the thermal behavior. Therefore, the incorporation of curcumin (0.54% *w*/*w*) did not have any drastic impact in the thermal behavior of the organogels. Similarly, the incorporation of β-carotene (0.01% *w*/*w*) in beeswax organogels did not influence their thermal parameters when beeswax concentration was 6% or 8% (*w*/*w*). In contrast, significantly higher values of melting enthalpy were found in organogels with 2% and 4% of beeswax as a consequence of the incorporation of β-carotene in the organogels, attributable to a reinforcing effect of the structure by the bioactive compound [16].

#### 3.2.4. X-ray Diffraction Analysis

Figure 3c shows the XRD patterns at 30 °C for the beeswax, OGCtrl and OGCur. Three sharp peaks were found for beeswax, one at low angle (corresponding to a 73.8 Å spacing) and two peaks at high angle (corresponding to 4.14 and 3.73 Å). For both organogels, the same peaks were found along with two diffuse peaks at low and high angle (3.3° and 19.7° in 2θ, respectively), in agreement with the isotropic pattern expected for the linseed oil in the organogel. The observed pattern was indicative of a β’ orthorhombic subcell structure, characterized by two strong wide-angle reflections at lattice spacings of 4.2–4.3 Å, corresponding to the (110) β’ reflection, and 3.7–3.9 Å, consistent with the (200) β’ reflection [47].

Figure 3d shows the temperature dependent XRD patterns obtained for OGCur as example (XRD patterns for beeswax and OGCtrl are shown in Figure S2a and S2b, respectively). The gel formation by cooling was observed at 53.5 °C and 52.4 °C in OGCtrl and OGCur, respectively, whereas the beeswax crystallized at 61.9 °C (Table 1). All these values are slightly higher than those obtained for T_1_ in the thermal profile for both beeswax and organogels (Table 1). This is because X-ray diffraction shows the structure formation as soon as it appears, whereas the crystallization temperature in DSC is defined as the point of intersection of the inflectional tangent at the beginning of the crystallization peak with the extrapolated baseline. The SAXD diffractograms of the beeswax and organogels showed the appearance of a sharp peak (73.8 Å in the beeswax and slightly shifted at 76.1 Å in OGCur and OGCtrl) corresponding to the (001) reflection peak, providing evidence of a lamellar packing. The d-value of the β (001)-reflection represents the thickness of the molecular layers [48]. This long-spacing peak at the SAXD 2θ angles has been described as a double-layer order structure made up of molecules with terminal polar groups (fatty acids, fatty alcohols) crystallizing with a head-to-head orientation [49]. As described by Kotel’nikova et al. [50], this peak at 73.3 Å corresponds primarily to the contribution of n-alkanes with 27, 29, 31 and 33 carbon atoms in different proportions and bilayered. A set of higher order (00n)-reflections indicates a regular, periodic structure in lamellar phases, and represents the periodical sequence of electronic density differences in multiple layers that was barely found in beeswax (Supplementary Figure S2a); indicating a lower order of the layer structure that is characteristic of mixtures of compounds with varying chain lengths [51,52].

#### 3.2.5. Rheological Analysis

**Figure 4:**
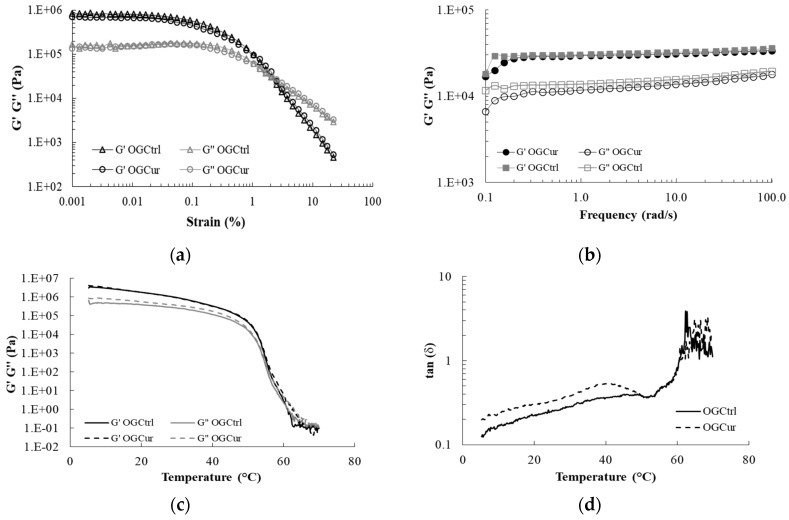
(**a**) G’ and G’’ as a function of strain during amplitude sweeps for OGCur and OGCtrl. (**b**) G’ and G’’ as a function of frequency for OGCur and OGCtrl. (**c**) G’ and G’’ as a function of temperature during heating from 5 °C to 70 °C for OGCur and OGCtrl. (**d**) Tan (δ) values a function of temperature during heating from 5 °C to 70 °C for OGCur and OGCtrl.

The shear strain was set at 0.01%, within the linear viscoelastic region (LVR), according to the strain amplitude sweeps run at 6.28 rad/s and 25 °C (Figure 4a). Storage modulus (G’) was over loss modulus (G’’) at small deformation both in OGCur and OGCtrl. The LVR was only found at very low strain, and the values of both moduli decreased with increasing strain, followed by a crossover between G’ and G’’. The incorporation of curcumin did not modify the structural stability of the organogel, as the critical strain at which G’ modulus dropped was around the same value of oscillation strain (around 0.1%, Figure 4a).

The mechanical spectra obtained from the frequency sweeps for OGCur and OGCtrl is shown in Figure 4b. No differences were found due to the incorporation of curcumin, and G’ values were higher than G’’ throughout the frequency sweep in both organogels, evidencing again a predominant elastic behavior. Furthermore, G’ was independent from the frequency applied in the range studied (0.1–100 rad/s), whereas only a slight increase in G’’ was observed with increasing frequency values, which indicated that the organogels support external forces in the range of frequency used. Tan δ was <1 but >0.1, indicating that both organogels behaved as a solid-like material with a weak gel character [10]. This predominant elastic behavior, due to the presence of a beeswax crystal network immobilizing the liquid linseed oil, has been reported for other beeswax organogels [10]. In contrast with these results, Martins et al. [16] found a change in the rheological properties of beeswax organogels when β-carotene was incorporated into the system. Thus, beeswax organogels with β-carotene showed higher G’ values than those without the bioactive compound, with predominance of elastic properties, attributed to the formation of a stronger crystal network when β-carotene was incorporated.

Figure 4 shows the changes in G’, G’’ (Figure 4c) and tan δ (Figure 4d) as a function of increasing temperature from 5 to 70 °C. A slight decrease of both G’ and G’’ was found in both organogels from 5 °C to 50 °C. Afterwards, both moduli drastically decreased with increasing temperature until 62 °C, indicating an important breakdown of the crystal network (G’ values close to 0 Pa). Furthermore, G’’ crossed over G’ and tan δ was >1 at 60.6 °C both in OGCur and OCCtrl, in agreement with the end of the melting event found in the MDSC thermogram (Figure 3a,b), indicating the complete melting of the beeswax and the transition from an elastic solid to a viscous liquid. In view of these results, it can be concluded that the incorporation of curcumin in the organogel formulation had no effect on the thermal stability of the organogels. This thermal behavior is expected for wax organogels, since most of the waxes have melting points between 60 and 70 °C [43]. Gómez-Estaca et al. [10] found a decrease of G’ values between 52 °C and 60 °C in beeswax organogels (10% *w*/*w*), formulated with a mixture of olive, linseed and fish oil, as a consequence of the melting of the organogelator. Martins et al. [53], found a sharp decrease of G’ and G’’ moduli in the range of temperatures 45–55 °C in the thermal profile of beeswax organogels.

#### 3.2.6. Long-Term Oxidation Study

**Figure 5:**
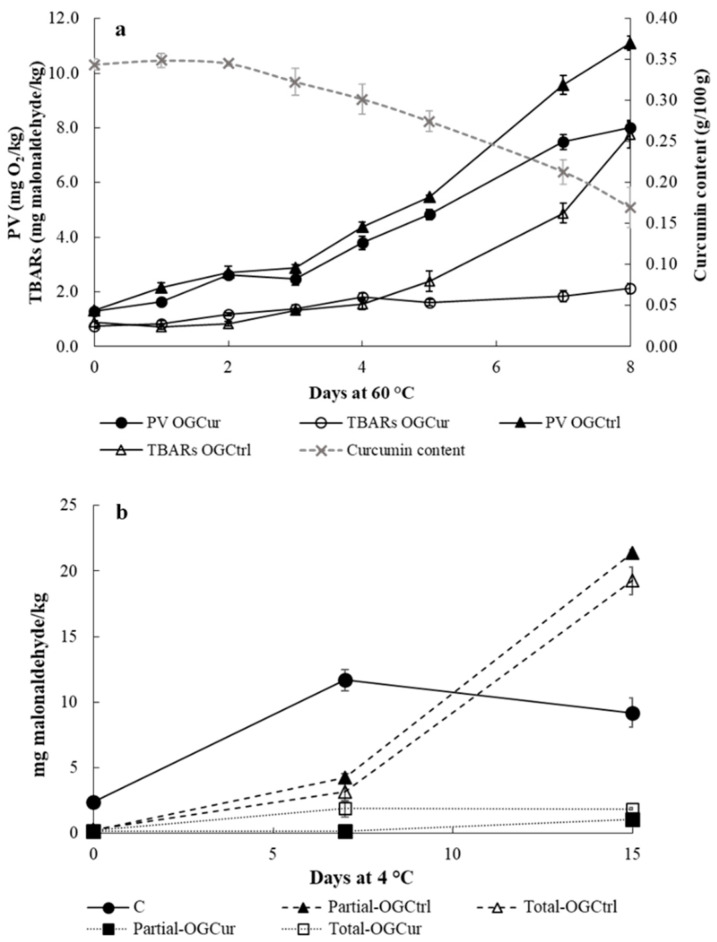
(**a**) Evolution of peroxide values (PV), TBARs values (TBARs) and curcumin content in OGCur and OGCtrl during accelerated oxidation conditions (60 °C). (**b**) TBARs values in pâtés with different lipid ingredients used in the formulation during chilled storage (C, pâté with pork backfat; Partial-OGCur, pâté with partial replacement of pork backfat by OGCur; Partial-OGCtrl, pâté with partial replacement of pork backfat by OGCtrl; Total-OGCur, pâté with total replacement of pork backfat by OGCur; Total-OGCtrl, pâté with total replacement of pork backfat by OGCtrl).

Figure 5a shows the evolution of peroxide value, TBARs and curcumin content for OGCur and OGCtrl during storage at 60 °C. Two zones can be distinguished in this graph (Figure 5a): during the first three 3 days, the curcumin content remained constant and there were not differences between OGCtrl and OGCur both in peroxide value and TBARs. In this zone, the absence of curcumin degradation can be explained due to the antioxidant effect of tocopherols. Linseed oil naturally contains α, γ, and δ-tocopherols, and the most abundant isomer is the γ-form (about 60% of the total content). The antioxidant activity of tocopherols has been attributed to their ability to donate hydrogen atoms to peroxyl radicals, preventing the oxidation of polyunsaturated fatty acids [54]. 

Contrary, the second zone (after four days) was characterized by a decrease in the curcumin content, where the addition of curcumin to the organogel (OGCur) showed a noticeable protective effect on both peroxide and MDA formation. The decrease in the curcumin content may be explained by the antioxidant activity of the curcumin. Two antioxidant mechanisms of curcumin have been suggested in the literature, donation of H atoms from the phenolic groups [35] and/or abstraction of H atoms from the central active CH_2_ group [55]. However, H atom donation from the phenolic groups of curcumin is the most suitable antioxidant mechanism in lipid matrices, as was reported for methyl linoleate [35]. The decrease in the curcumin content in the second zone suggests that tocopherols were depleted in the first zone. Similarly, Beddows et al. [56] reported that curcumin had an antioxidant effect in sunflower oil heated at 105 °C, but it did not improve the preservation of α-tocopherol. The incorporation of curcumin in corn oil organogels based on β-sitosterol and lecithin increased the oxidative stability at 60 °C, measured by peroxide value and *p*-anisidine, compared to a curcumin-free organogel [23]. However, the degradation of curcumin was not addressed in that study.

Although an accelerated oxidation study of the organogels with and without curcumin was performed at 60 °C for comparative purposes with other studies, this storage temperature was above the melting temperature of the organogels (Table 1). In this case, organogels had liquid-like viscous behavior similar to liquid oils, where both the oxygen diffusion coefficient and the movement of reactants increase, increasing the oxidation rate of the polyunsaturated fatty acids of linseed oil [23,57].

### 3.3. Oxidative Stability of Spreadable Meat Products (pâté)

The oxidative stability of pâtés was evaluated through the determination of TBARs. Figure 5b shows the TBARs values obtained for the different pâtés during storage at 4 °C. Pâtés are meat products very prone to lipid oxidation, since they are products with high content of fat and low content of natural antioxidants [58]. Furthermore, pâtés are highly processed products that are subjected to grinding and cooking that can accelerate the lipid oxidation [15]. At the beginning of the chilled storage, all the samples formulated with OGCur or OGCtrl (partial-OGCur, partial-OGCtrl, total-OGCur and total-OGCtrl) showed significantly lower (*p* < 0.05) TBARs values than the control samples (C, formulated with pork backfat), which may be attributed to the MDA formation during the thermal treatment due to the relatively high content of linoleic acid (17.06 ± 0.11%; Table S3) and the absence of naturally occurring antioxidants in C. Although TBARs values significantly (*p* < 0.05) increased in all the samples throughout the storage, differences in the formation of MDA were found according to the lipid ingredients used in the formulation that may be attributed to differences in the fatty acid composition, which is one the main factors affecting the lipid oxidation [15], and to the presence of curcumin. All the pâtés formulated with organogels (Partial-OGCur, Partial-OGCtrl, Total-OGCur and Total-OGCtrl) showed lower (*p* < 0.05) TBARs values than C at day 7 of storage, and those formulated with OGCur showed the lowest values (0.14 ± 0.03 and 1.89 ± 0.64 mg MDA/kg for Partial-OGCur and Total-OGCur, respectively). At day 15 of storage, a sharp increase of TBARs values was found in the pâtés formulated with OGCtrl (Partial-OGCtrl and Total-OGCtrl), reaching values close to 20 mg MDA/kg. This increase in TBARs values may be attributed to the high percentage of α-linolenic acid in linseed oil, very prone to lipid oxidation, together with the depletion of its naturally occurring antioxidants. Similarly, Moghtadei et al. [13] reported that lipid oxidation was higher in beef hamburgers formulated with 25% and 50% of sesame beeswax organogels than in control hamburgers with 100% beef fat during frozen storage. Partial-OGCur and Total-OGCur samples showed the lowest TBARs values at day 15 of storage, denoting a protective effect of curcumin against lipid oxidation, especially in pâtés with partial replacement (Partial-OGCur), where the total content of linoleic acid plus linolenic acid was around 17% lower than in pâtés with total replacement of pork backfat (Total-OGCur; Table S3). Both pâtés with linseed organogels with curcumin showed TBARs values below 2 mg MDA/kg at day 15 of storage, which has been stablished as limit in which there is not rancidity in meat and meat products in some studies [15]. In other meat products (pork burgers), Gómez-Estaca et al. [12] reported that curcumin effectively reduced lipid oxidation during chilled storage when it was incorporated in the formulation of high PUFA organogels based on beeswax and ethylcellulose.

## 4. Conclusions

Linseed oil beeswax-based organogels with mechanical properties similar to pork backfat, high oil binding capacity and oxidative stability were designed in this study. The incorporation of curcumin in the organogel did not affect the arrangement of the crystal network (β’ orthorhombic subcell structure), nor the mechanical, thermal, or rheological properties (predominant elastic behavior), suggesting that curcumin-triglyceride interactions were predominant on curcumin-beeswax ones. Furthermore, curcumin effectively improved the oxidative stability of the organogels and pâtés when these organogels were used as a lipidic ingredient to partially or totally replace pork backfat, although other oxidation indicators in addition to TBARs should be also evaluated in pâtés. These organogels based on linseed oil can be used as an ingredient for the replacement of animal fat in the development of healthier pâtés, rich in omega-3 polyunsaturated fatty acids, where the incorporation of antioxidants (curcumin) in the formulation should be considered for their successful application.

## Figures and Tables

**Table 1:** Upper half: Characteristics of the linseed organogels with curcumin obtained under optimal conditions (OGCur) and control linseed organogels without curcumin (OGCtrl). Lower half: MSDC (total heat flow) data for beeswax, OGCur and OGCtrl from Figure 3a.

**Parameters**				**OGCur**			**OGCtrl**		
Curcumin content (g/100 g)				0.34 ± 0.01			-		
Mechanical strength (N)				19.51 ± 0.77 ^a^			19.10 ± 1.16 ^a^		
OBC (%) day 1				91.4 ± 1.5 ^a^			90.4 ± 1.4 ^a^		
OBC (%) day 7				80.4 ± 3.7 ^a^			80.0 ± 1.2 ^a^		
IP (h)				9.80 ± 0.57 ^b^			4.37 ± 0.26 ^a^		
Protection factor				2.24 ± 0.13			-		
**Samples**	**T_TAGonset_**	**T_TAG_**	**ΔH_TAG_**	**T_onset_**	**T_1_**	**T_2_**	**T_3_**	**ΔH_t_**	**T_gel_X-ray_**
Beeswax heating	-	-	-	41.2	61.7	60.2	51.1	161.9	-
Beeswax cooling	-	-	-	63.2	60.2	58.7	49.9	153.5	61.9
OGCtrl heating	−29.1	−23.8	2.8	35.6	52.7	51	-	17.8	-
OGCtrl cooling	−9.4	−18.6	1.1	54.5	51	42.6	37.3	16.5	53.5
OGCur heating	−28.7	−23.8	2.5	35.7	52.7	51	-	17.1	-
OGCur cooling	−9.3	−17.9	1	54.4	51.4	43.3	37.7	17.8	52.4

IP: Induction period; OBC: Oil binding capacity; OGCur: organogel obtained under optimal conditions; OGCtrl: organogel without curcumin and the same beeswax concentration. Different lowercase letters in the same row mean significant differences (*p* < 0.05) between OGCur and OGCtrl. T_TAG_: peak temperature for the triglyceride melting/crystallization transition; ΔH_TAG_: enthalpy for the triglyceride melting/crystallization transition; T_1_, T_2_ and T_3_: peak temperatures for the wax melting/crystallization transitions; ΔH_t_: total area for wax melting/crystallization transition (T_1_, T_2_ and T_3_ peaks); T_gel_X-ray_: wax crystallization temperature found in XRD patterns.

## Supplementary Materials

The following are available online at https://www.mdpi.com/2076-3921/9/8/735/s1, Table S1: Formulation of pâtés (g/kg) with different lipid ingredients (pork backfat, OGCur or OGCtrl). Table S2: Experimental design for the preparation of linseed organogels with curcumin. ANOVA for mechanical force, OBC and IP of linseed organogels. Figure S1: MDSC thermograms for beeswax, first heating and first cooling from −30 °C to 90 °C. a) Total heat flow, with the temperature for the main peaks and b) Total heat flow and comparison with its reversible and nonreversible components (note the axis scale). Figure S2: Temperature dependent XRD patterns during cooling from the isotropic state for (a) beeswax and (b) OGCtrl. Table S3: Fatty acid profile of pâtés formulated with different lipid ingredients.
